# About the Irrationality of the Health Field

**DOI:** 10.3389/fpsyt.2018.00126

**Published:** 2018-04-10

**Authors:** Daniel David

**Affiliations:** ^1^Department of Clinical Psychology and Psychotherapy, International Institute for Advanced Study in Psychotherapy and Applied Mental Health, Babeş-Boylai University, Cluj-Napoca, Romania; ^2^Department of Population Health Sciences and Policy, Icahn School of Medicine at Mount Sinai, New York, NY, United States

**Keywords:** evidence-based practice, psychotherapy, evaluative framework, etiopathogenetic theory, mechanism of change

## The Problem

Looking back through the current lens, along history, the health field has been the stage for various scientific (including also the philosophical-based approaches before modern science), pseudo-scientific (e.g., astrology), and non-scientific (e.g., voodoo) practices/interventions. Typically, a health intervention has a practical component (i.e., the treatment) and a theoretical one (i.e., the theory/mechanism of change underlying the efficacy/effectiveness of the treatment).

As it is well-known in the field, in the modern period (particularly starting with the nineteenth century), the health field embraced the etiopathogenetic framework. This framework is based on the following logic: if an etiopathogenetic factor (e.g., bacteria) generates/is causally involved in clinical symptoms, then the treatment should be focused on changing the etiopathogenetic factor (e.g., by using antibiotics). Formalized, this means that if A (e.g., bacteria) generates B (e.g., symptoms), in order to change B (e.g., symptoms), one needs to change A (e.g., bacteria). However, from a logical point of view, this is not the strongest clinical strategy, because it is based on faulty logic (i.e., *denying the antecedent*): non-A (e.g., non-bacteria) does not necessary imply non-B (e.g., no symptoms); of course, at the same time, B (e.g., symptoms) does not always imply A (e.g., bacteria) (*affirming the consequent*). Only *modus tollens*—A (if bacteria) implies B (then symptoms)—and *modus pones*—non-B (no symptoms) implies non-A (no bacteria)—are valid logical arguments. The effects of interventions based on etiopathogenetic theories were mostly assessed by case observations and/or controlled studies. However, few of these controlled studies were randomized trials, which can really test the effects of non-A rigorously. Therefore, although the etiopathogenetic framework increased the presence of science in the health field (e.g., by encouraging clinical research into etiopathogenetic theories)—gradually pushing pseudo- and non-scientific practices out of the health mainstream-, it still encouraged a strong reliance on expert consensus in establishing the gold standard treatments (e.g., as randomized trials were less available), which was prone to strong subjectivity. Having said that, one might note that in the mental health field etiopathogenetic mechanisms can be often multifactorial (e.g., stressing activating events × irrational beliefs), rather than unifactorial. However, while the content of “A” can be, indeed, more or less complex, the above mentioned logical framework remains valid.

Therefore, beginning with the middle/end of the twentieth century, the health field embraced the evidence-based framework, having randomized clinical trials as its core. Indeed, starting from the etiopathogenetic framework, the clinical utility of non-A (e.g., no-bacteria/no activating events × irrational beliefs) should be tested in randomized clinical trials, thus exploring statistically the probability that non-A (e.g., no-bacteria/no activating events × irrational beliefs) generates non-B (no symptoms). However, by focusing too much on testing the practical component (i.e., the treatment), the health field unwisely ignored the underlying theories of various treatments. Such an approach is limited and very problematic, as it allows pseudo-scientific and/or non-scientific approaches to get back into the health care system. Indeed, such approaches can do better in clinical trials than certain weaker control conditions (e.g., no intervention, wait list—which sometimes can even have a nocebo effect, and less active placebo), potentially confounding their more active placebo clinical effect with a specific clinical effect. This excessive focus on *what works* (in classical evidence-based framework), not also on *how and why it works* (e.g., the underlying etiopathogenetic theory and/or mechanism of change) introduced several major weaknesses and risks into the health field. Let us briefly examine the most important ones.

### The Proliferation of Pseudo- and Non-Scientific Approaches in the Evidence-Based Health System—The Babel Tower

Recently, various funding agencies and publications have increasingly focused on the efficacy/effectiveness of health interventions that do not rely on scientific theories, but on pseudo-scientific (e.g., the healing role of “universal energy”) and/or non-scientific (e.g., religious-based such as Yoga) ones. Simply said, by scientific theories we mean research-supported theories in a testable framework [see for details (1, 2)]. To delineate them from standard/conventional medicine, these interventions were often called complementary/alternative/unconventional medicine. There is nothing wrong with such practices/interventions and they can be supported, as long as the consumer is made aware of the fact that their underlying theories are untested (or even untestable). For example, if one does not address the underlying Yoga theory and its relation with the clinical condition for which Yoga is tested as a treatment—be it the etiopatogenetic theory or the mechanism of change responsible for symptomatic improvements-, one is left with the possibility that improvements in patients’ symptoms may be attributed to past life mechanisms and/or to attaining various levels of consciousness (never tested or even untestable), rather than to other mainstream and research-supported mechanisms (e.g., placebo, behavioral activation for physical exercise). And the Yoga example could be applied to many complementary/alternative approaches.

One could argue that such approaches are asked for, supported, and widely used by consumers and society. Indeed, the National Center for Complementary and Integrative Health (NCCIH) of the National Institute for Health adopts such a seductive and apparently socially responsible logic, by arguing that because people largely use such practices in their life, it is in their best interested that NCCIH funds tests of their efficacy/effectiveness and safety. Moreover, this strategy is even described as a sign of respect for consumers’ beliefs and values. Finally, such approaches are often presented as “sexy,” while various scientific approaches are deemed “old.” Well, if science can build on common sense, it is great, and we should follow this path, which is already supported by the NCCIH. However, there are moments when science should educate and change common sense, especially when it is deluded in some sense. This is how humankind has evolved and built civilizations. For example, as it is well-known in the health literature, not long ago people thought that Malaria was caused by “bad air,” rather than by the infectious agent we know today. Moreover, if we had run a trial testing the efficacy of closing doors/windows in blocking “bad air” and thus preventing/reducing the symptoms of Malaria, it might have proven efficient/efficacious compared to a control condition. Would this approach have been good science? Finally, many medical interventions for various clinical conditions might not look “sexy” at first and they could be already quite “old.” However, they become attractive and traditional (not only old) by often saving patients’ life!

Complementary/alternative treatments should be called as such until we test them. If they pass the test for both treatment and theory, then they become standard health practice. If they pass the test for efficacy/effectiveness of the treatment, but no for theory, then they can be used in the health field, but reinterpreted based on a research-supported theory. For example, isolating yourself—by closing doors/windows—during a malaria outbreak can help you, but not because you avoid “bad air,” but because you avoid the etiopathogenetic agent. Similarly, Yoga might be just a useful physical and/or behavioral activation exercise.

Unfortunately, in the current logic of the evidence-based framework, many funding agencies and/or publishers do not pay enough attention to the underlying theories of such complementary/alternative practices, thus leaving the door open for the contamination of the health field by pseudo- and non-scientific practices and bringing them back in the mainstream.

### Stimulation of Regressive Rather Than Progressive Programs—Freezing in the Moment and the Return of the “Old/Repressed”

In the current evidence-based framework, more and more clinical trials are designed in equivalence and/or non-inferiority, rather than in superiority terms. This somehow suggests that we might have more and more difficulties in finding new treatments much better than the ones available. Therefore, we look for new treatments, which although no more efficacious/effective than existing ones, might bring other benefits (e.g., they are more cost-effective, more accessible). And this is a good and legitimate approach. However, sometimes, such approaches can be ill-used.

For example, in the mental health field, this approach, rather than leading to new potentially more cost-effective treatments, has resulted in the reactivation of old treatments (e.g., psychoanalytically based), which have no added benefits compared to existing evidence-based interventions from an efficacy/effectiveness point of view [see, for example, Ref. (3)] and which are even based on questionable/controversial theories (see, for example, the controversial status of the psychoanalytical theory for panic disorder at Division 12 of the American Psychological Association: https://www.div12.org/psychological-treatments/treatments/psychoanalytic-treatment-for-panic-disorder/). Indeed, research-support for the theory underlying a treatment cannot be inferred based on the efficacy/effectiveness of the treatment derived from the theory, as this reflects the faulty logic of *affirming the consequent*, but the theory should be tested directly. Finally, equivalence and non-inferiority designs are not transitive. If a treatment proves equivalent or non-inferior compared to the gold standard, it does not mean that it can be used as a gold standard itself, but it should first pass independently the same tests as the gold standard (i.e., if B is equivalent to A, and C is equivalent to B, it does mean that C is equivalent to A) (4).

People often confuse the gold standard status of a health practice with the ideal status. An evidence-based gold standard status for a treatment just means that the treatment is the best we have, not that the treatment is the best we can ever have. For example [see for more details the analysis of Ref. (4)], in the mental health field, various colleagues [e.g., Ref. (5)] challenged the gold standard status of cognitive-behavioral therapy (CBT), pointing to the low number of high-quality studies and to weak control conditions, thus arguing for a plurality of psychotherapies. While it is true that CBT has been assessed in many low-quality studies and/or with weak control conditions, no other psychotherapy has more studies of high quality and strong control and/or a better research-supported underlying theory/mechanisms of change. For example, according to Cuijpers et al. (6), about 54% of the trials for depression and about 20% of the trials for anxiety meet the criteria for a strong comparison (i.e., pill placebo or TAU), and 17% of the total trials for depression and anxiety are of high quality. Other forms of psychotherapy do not even come close to these numbers [see Ref. (7) for psychodynamic therapy in depression and (8) for psychodynamic therapy in anxiety]. However, rather than suggesting to improve research in the CBT field and/or even to look for new more effective/efficacious practices, beyond CBT, various colleagues—in the name of a seductive, but still poorly conceptualized “plurality”—argue for the reactivation of old treatments (e.g., psychoanalytically based), especially those which can be proved equivalent to CBT, even if, as mentioned above, they often (a) have a controversial theory/mechanisms of change; (b) have a lower number of studies of high quality and of strong control compared to CBT; and/or (c) are proposed as gold standard based on the misunderstanding of transitivity. Moreover, various old treatments are reactivated even in weaker scientific frameworks, by being compared to no intervention/waitlist/or other weak control conditions (again potentially confounding their active placebo clinical effect with a specific clinical effect), often ignoring their underlying theory/mechanisms of change.

## Solution: Back to Rationality and Wisdom

In an effort to correct some of these major weaknesses and risks, David and Montgomery (1) argued for a reconceptualization of the evidence-based framework, in which both the treatment and its underlying theory are validated [see Table 1; see also Ref. (9) for an example regarding the application of the new system for various clinical conditions]. It is a modern revival of the eighteenth century approach of the French Academy of Science in dealing with pseudo-science such as mesmerism (i.e., an approach based on a “universal energy,” named *animal magnetism*).

**Table 1:** A new evaluative framework of psychotherapies [reproduced with permission after (1)].

Therapeutic package	Theory		
	Well supported^a^	Equivocal: no, preliminary, or mixed data^b^	Strong contradictory evidence^c^
Well supported^d^	Category I	Category II	Category V
Equivocal: no, preliminary, or mixed data^b^	Category III	Category IV	Category VII
Strong contradictory evidence^c^	Category VI	Category VIII	Category IX

*^a^Well-supported theories are defined as those with evidence base on (1) experimental studies (and sometimes additional/adjunctive correlational studies) and/or (2) component analyses, patient–treatment interactions, and/or mediation/moderation analyses in complex clinical trials (CCTs). Thus, the theory can be tested independently of its therapeutic package (e.g., in experimental studies and sometimes additional/adjunctive correlational studies) and/or during a CCT. “Well supported” within this framework means that a theory has been empirically supported in at least two rigorous studies, by two different investigators or investigating teams*.

*^b^Equivocal evidence for the therapeutic package and/or theory means no data (data not yet collected), preliminary data (there are collected data, be it supporting or contradictory, but they do not fit the minimum standards), or mixed data (there are both supporting and contradictory evidence)*.

*^c^Strong contradictory evidence for the therapeutic package and/or theory means that it has been empirically invalidated in at least two rigorous studies, by two different investigators or investigating teams*.

*^d^Well-supported therapeutic packages are defined as those with randomized clinical trials (or equivalent) evidence of their efficacy (absolute, relative, and/or specific) and/or effectiveness. “Well supported” within this framework means that a package has been empirically supported in at least two rigorous studies, by two different investigators or investigating teams*.

*The darker backgrounds (Categories V–IX) signify pseudo-scientifically oriented psychotherapies (POPs); the core of the POPs is Category IX. The lighter backgrounds (Categories I–IV) signify scientifically oriented psychotherapies (SOPs); the core of the SOPs is Category I. Depending on the progress of research, a psychotherapy could move from one category to another*.

In this framework, the underlying theory can be related to the etiopathogenetic mechanisms of the clinical condition (and thus the treatment is an etiopathogenetic one) or to other mechanisms (and thus the treatment is often a symptomatic one). Typically, a new treatment starts in Category IV—experimental/investigational treatment—and then, depending on the data on both theory and treatment, progresses or regresses into other categories. The gold standard would be represented by Category I. Categories II and V should be worked on to find the research-supported underlying theory (e.g., most complementary/alternative treatments shown to work would probably fit these categories). For categories III and IV, we should work on better deriving treatments from research-supported theories. Categories VII and VIII should be investigated programmatically (e.g., in large scale powered clinical trials/in crucial experiments) to understand why the mixed results have emerged and what the real status of the treatment and/or theory is. Finally, Category IX should be preserved for the history of the field, considering that, if used, it would represent pseudo-science.

Summarizing, the health field is in a dangerous phase, as many pseudo-scientific, non-scientific, and/or outdated treatments are more and more vocal and invasive. The health field seems to behave in the logic of regressive scientific programs, by relaxing the scientific criteria (e.g., by minimizing the underlying theory of various treatments, thus stimulating pseudo- and non-scientific approaches) and/or reactivating outdated treatments (e.g., by trying to show that they are better than no intervention/waitlist/or other weak control conditions and/or equivalent/non-inferior to already existing treatments), rather than developing new more efficacious treatments. For a healthy development, in the benefit of science and society/consumer, the current evidence-based framework must reconsider and implement the etiopathogenetic tradition (i.e., paying more attention to the theory/mechanisms underlying a clinical condition and its treatment) and favor superiority designs (with strong control conditions relating to the best-established treatment). Equivalence/non-inferiority designs should only be considered when other clear benefits of the investigated treatment compared to established treatments, beyond the similarity in efficacy/effectiveness, can be expected. Such benefits should be represented by higher cost-effectiveness, shorter duration, lower dropout rate, better accessibility and acceptability, and/or less side effects (or when the comparison with the placebo control is not ethical). In addition, patient preferences are important if they could be clearly documented, but remembering that patients can and should be educated to support a knowledge-based society (e.g., preferences/views based on controversial/invalidated scientific theories and/or practices should be educated). Only by endorsing such a philosophy, we can make sure that progressive rather than regressive programs, in the benefit of humankind, will guide the health field.

## Author Contributions

DD substantially contributed to the conception of the work, writing the manuscript. DD approves the submitted version of the manuscript and agrees to be accountable for all aspects of the work.

## Conflict of Interest Statement

DD is the director for research at the Albert Ellis Institute and a diplomate in CBT at the Academy of Cognitive Therapy, both organizations strongly promoting evidence-based approaches in the health field.
